# 2616. Effect of Vitamin D on Clinical Outcomes in Patients Hospitalized with Influenza: A Retrospective Study

**DOI:** 10.1093/ofid/ofad500.2229

**Published:** 2023-11-27

**Authors:** Suk Yin Chan-Colenbrander, Qi Wang

**Affiliations:** University of Minnesota, Minneapolis, Minnesota; University of Minnesota, Minneapolis, Minnesota

## Abstract

**Background:**

Seasonal influenza infection causes significant morbidity and mortality worldwide. This study evaluates the effect of vitamin D on clinical outcomes in patients hospitalized with influenza infection.

**Methods:**

We performed a retrospective chart review of patients (age 18 years and older) admitted between January 1, 2016, and December 31, 2019, to the University of Minnesota Medical Center, who were tested positive for influenza.

Data were summarized using median and interquartile range (IQR) for continuous variables, frequency and percentage for categorical variables. Groups were compared using two-sample Wilcoxon test for continuous variables and Chi-square test (or Fisher's exact test for sparse data) for categorical variables.

**Results:**

Out of 320 patients tested positive for influenza, 180 were vaccinated against influenza. The median age of those tested positive in the vaccinated group was 64 (IQR 49-74) and the unvaccinated, 58 (IQR 35-71) p=0.0074. 97.8% of the vaccinated and 94.3% of the unvaccinated patients received oseltamivir. Significantly more unvaccinated patients were admitted to the intensive care unit (ICU) (p=0.0029) and required ventilatory support (p=0.0097) compared to those who were vaccinated.

55% of the vaccinated and 36.4% of the unvaccinated patients were on Vitamin D (p=0.001). Vitamin D median level was significantly higher in the vaccinated group, 35 (IQR 22-46) compared to 24 (IQR 15-36) in the unvaccinated group (p=0.0001).

Vitamin D levels did not significantly affect the length of hospital stay, ICU admission, respiratory or cardiovascular complications, hospital survival, survival at 1- and 3-years post hospitalization in both the vaccinated and unvaccinated groups.

**Conclusion:**

Vitamin D levels did not significantly affect the clinical outcomes of patients hospitalized with influenza.

**Disclosures:**

**All Authors**: No reported disclosures

